# Mechanistically Explainable AI Model for Predicting Synergistic Cancer Therapy Combinations

**DOI:** 10.3390/curroncol32100548

**Published:** 2025-09-30

**Authors:** Han Si, Sanyam Kumar, Sneh Lata, Arshad Ahmad, Saurav Ghosh, Karen Stephansen, Deepti Nagarkar, Eda Zhou, Brandon W. Higgs

**Affiliations:** 1 Translational Data Sciences, Genmab, Princeton, NJ 08536, USA; 2 Commercial Data Sciences, Genmab, Princeton, NJ 08536, USA; 3 Global IT and Digital, Genmab, Princeton, NJ 08536, USA; 4 R&D Strategic Initiatives, Genmab, Princeton, NJ 08536, USA; 5 Lamini, Menlo Park, CA 94025, USA

**Keywords:** oncology, Large Language Model (LLM), drug combination, knowledge graph, retrieval-augmented generation (RAG), clinical trial

## Abstract

This study introduces a Large Language Model (LLM)-based framework that combines drug combination data with a knowledge graph to predict synergistic oncology drug combinations with mechanistic insights. Using a retrieval-augmented generation (RAG) approach, over 50,000 in vitro drug pair assay results and 1631 human clinical trial and preclinical test entries were integrated to enhance predictive accuracy and explainability. Validation achieved an F1 score of 0.80, demonstrating the framework’s potential to streamline drug discovery and improve translational strategies in cancer treatment.

## Simple Summary

Cancer treatment often uses drug combinations to combat tumors more effectively, but identifying synergistic pairs is challenging. This study introduces an AI framework using Large Language Models with retrieval-augmented generation to predict and explain these pairs, achieving very good performance in validation. The approach could expedite drug discovery and shape policies promoting AI in oncology for better patient outcomes.

## 1. Introduction

The complexity and adaptability of cancer make single-agent treatments less effective, as tumors quickly develop resistance [1]. Drug combinations address this by targeting multiple pathways concurrently, reducing resistance and often enhancing effectiveness [2]. For instance, pairing immune checkpoint inhibitors with chemotherapy boosts immune response while targeting tumor cells [3,4]. Similarly, combining BRAF and MEK inhibitors has improved outcomes in melanoma [5,6].

Over the past two decades, in silico methods for predicting drug synergy have advanced drug combination discovery, reducing the need for resource-intensive lab experiments [7]. Early models integrated transcriptomic and chemical-genomic data [8], while initiatives like the NCI-DREAM Challenge spurred models such as DeepSynergy [9,10]. Recently, transformer-based models trained on large-scale in vitro datasets like CancerGPT have further improved predictive accuracy [11].

Here we develop and evaluate an LLM-plus-knowledge-graph framework to predict clinical synergy of oncology drug combinations. Unlike prior cell-line-focused methods, our target is human trial performance, and sometimes predictions are modeled to be restricted to clinically biomarker-defined subcohorts and validated against late-phase endpoints (mostly phase III).

## 2. Materials and Methods

### 2.1. PubMed Data Collection

Relevant publications were accessed through the NCBI Entrez E-utilities API, dynamically generating queries using drug names and journal titles to target articles on specific drug combinations. Retrieved data were processed using BeautifulSoup v4.12.3. OpenAI GPT-4 extracted biomedical data from abstracts and full texts. Tailored prompts facilitated three types of extraction: (1) abstract summarization, highlighting tumor type and drug efficacy, (2) drug target identification, and (3) tumor type and clinical trial outcomes. Outputs were validated by manual review and cross-referencing with DrugBank to ensure alignment with established biomedical data. Error handling was integrated for API call robustness.

### 2.2. Clinical Trial Data from Citeline Trialtrove

Additional phase III clinical trial data were obtained from Citeline Trialtrove, focusing on trial IDs, phases, and outcomes. GPT-4 identified drug targets, categorizing trial outcomes as positive, negative, or unknown. The total set of unique drug modality combinations and clinical trial numbers are provided in the Supplementary Data.

### 2.3. In Vitro Drug Combination Screening Database

DrugComboDb [12] was downloaded (release date: 31 May 2019), inclusive of >50,000 drug combinations across 51 cancer cell lines; synergy scores were categorized using ZIP, HSA, Loewe, and BLISS scores (synergy if ZIP > 10 or any other 2 scores > 10; antagonism if ZIP < 10 or any other 2 scores < 10; inconclusive otherwise). Drug names were merged with CancerDrugsDB to associate protein targets with each drug, and the Cancer Cell Line Encyclopedia (CCLE) [13] was used to associate cell lines with the tumor from which they were derived.

### 2.4. Knowledge Graph for Biological Explainability

A biological knowledge graph—PrimeKG [14] containing proteins, pathways, diseases, and drugs (7080 diseases with 4,050,249 relationships) was used to enhance model explainability.

### 2.5. Model Architecture

**Figure 1:**
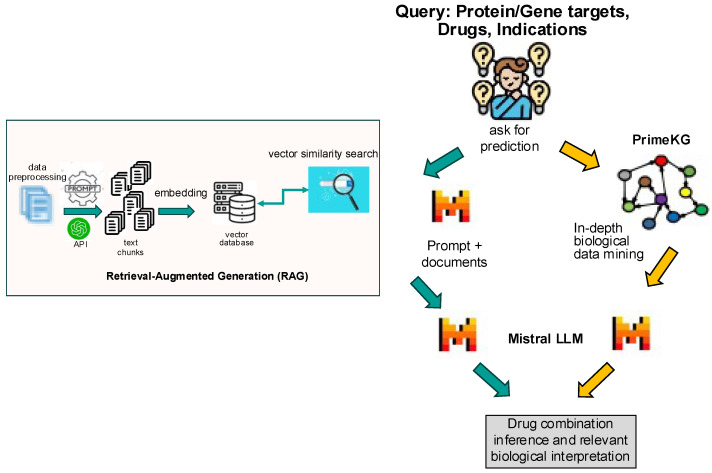
Model architecture illustrating the input data flow as RAG into a vector database that was queried by the user [Drugs, Gene/Protein targets, Indications] using the LLM and the knowledge graph to provide a prediction result with a biological rationale for the drug combination in the specific indication.

The model architecture consisted of a hybrid framework integrating an LLM with a knowledge graph (Figure 1). The first step was to implement retrieval-augmented generation (RAG): a corpus of drug treatment data from Trialtrove, publication summaries, and DrugComboDb were embedded into a text-based vector database. This RAG served as the foundation for the retrieval step, ensuring that the model had access to high-quality, relevant data. Next was to craft prompts to harness the full potential of the LLM, guiding it to focus on accurate drug combination predictions using the retrieved information from the RAG; each prompt aligned with the specific nuances of drug combinations in the query, ensuring that the output was both precise and insightful. The final step utilized the knowledge graph, which enriched the model’s predictions by anchoring on biological and disease context. By linking drug–target interactions to known disease biology, the knowledge graph enhanced interpretability, making the model’s predictions not only mere predictions but also providing biologically informed insights.

### 2.6. Software and Evaluation Metrics

Software licensed by Lamini (Menlo Park, CA, USA) was used for model implementation. Mistral v0.2 was the LLM in the model architecture. Model performance evaluation included standard classification metrics such as precision, recall, F1 score, and accuracy. Predictions were also assessed according to biological plausibility based on known mechanisms of action from the literature.

## 3. Results

Despite advancements, translating animal and cell line model findings to human trials remains challenging, particularly in providing mechanistic explanations for drug combinations [15]. Our study builds on prior methods with a new framework integrating phase 1–3 clinical trial outcomes, in vitro screens, and a biological knowledge graph (PrimeKG) to improve mechanistic insights.

By combining 1631 human study results across 723 drug modality combinations (Supplemental Figure S1) and 50,000 in vitro screens into a RAG model [16] with entity relationships (Figure 1), our approach offers biologically grounded, interpretable predictions to improve discovery and forward/reverse translational stages of clinical development.

**Figure 2:**
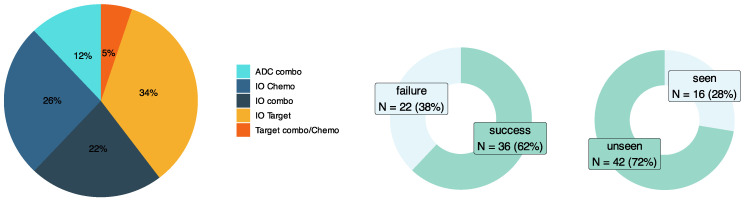
Distribution of manually curated validation set. Graphs show breakdown of drug combination modalities, success/failure status of clinical trials, and whether cases were seen/unseen by RAG. Note: the sum of percentages may not equal 100% due to rounding.

**Figure 3:**
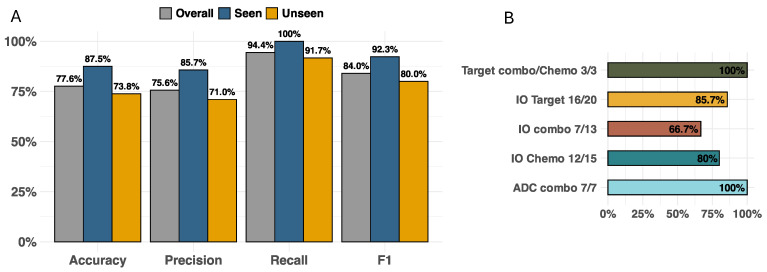
Model performance across all drug modalities in (**A**) overall, RAG-seen, and RAG-unseen cases and (**B**) F1 scores separated by drug modality combination in all cases.

A validation set inclusive of drug combinations and tumor types both seen and unseen by the input RAG was manually curated (Figure 2). This dataset consisted of 58 test cases exclusively from clinical trials across a diverse set of tumor types, patient genetic subtypes, and oncology drug modalities involving combinations of immuno-oncology (IO) therapies, targeted therapies, antibody–drug conjugates (ADCs), and chemotherapies. Figure 3A illustrates the model accuracy, precision, recall, and F1 score for this validation dataset with test cases seen and unseen by the RAG (overall, N = 58; seen, N = 16; and unseen, N = 42). Across all drug modalities, the model showed an overall accuracy of 77.6%, with accuracies of 87.5% for seen cases and 73.8% for unseen cases. The F1 scores of overall, seen, and unseen test cases were 84%, 92.3%, and 80%, respectively. For F1 scores across drug modalities (Figure 3B), ADC agents in combination with other therapies performed the best (100%), which tied with the performance of targeted therapies combined with either other targeted or chemotherapy agents (100%), while IO-IO combinations performed the worst (66.7%). To illustrate the mechanistic explainability of the predictions, a few examples unseen by the model are provided below with additional examples in the Supplementary Data.

The model successfully predicted the combination of atezolizumab (anti-PD-L1) and cobimetinib (MEK1/2 inhibitor) to not be effective in metastatic colorectal cancer (CRC), consistent with the phase 3 IMblaze370 trial, where the combination failed to improve overall survival compared to regorafenib [17]. Beyond accuracy, the model provided detailed mechanistic reasoning, abbreviated here: “Atezolizumab is an anti-PD-L1 monoclonal antibody that blocks this interaction, thereby restoring T-cell function and enhancing the immune response against cancer cells. Cobimetinib, on the other hand, is a MEK1/2 inhibitor that blocks the MAPK signaling pathway, which is involved in cell growth and survival. While both drugs have shown activity in various cancers, there is no definitive data on their synergy in locally advanced or metastatic CRC. In fact, there is evidence suggesting that MEK inhibitors may decrease the efficacy of PD-1 blockade by increasing the expression of PD-L1 in some cases. Therefore, it is unlikely that the combination of atezolizumab and cobimetinib would have a synergistic effect in treating locally advanced or metastatic CRC.” This prediction aligned with clinical outcome, highlighting the model’s mechanistic explanation. MEK inhibition can upregulate PD-L1 [18,19], potentially increasing targets for anti-PD(L)1 therapy; however, this may also promote an immunosuppressive environment, thus explaining the lack of synergy in CRC.

A true positive prediction involved combining mirvetuximab soravtansine (anti-FOLR1 + tubulin inhibitor) with bevacizumab (anti-VEGFA) in platinum-resistant ovarian cancer. An abbreviated excerpt of the model’s rationale highlights complementary mechanisms: “…distinct mechanisms of actions targeting different molecular pathways involved in cancer progression…By targeting FOLR1, mirvetuximab soravtansine can disrupt the folate metabolism pathway and inhibit cell division, making the cancer cells more susceptible to chemotherapy…By inhibiting VEGF, bevacizumab can prevent the formation of new blood vessels, starving the tumor of nutrients and oxygen, and enhancing the efficacy of chemotherapy. The combination of these two drugs can lead to a synergistic effect by targeting different molecular pathways and enhancing the overall therapeutic effect against platinum-resistant ovarian cancer”. This synergy prediction demonstrates its potential to guide treatment in resistant tumors.

While the model performed well overall, IO-IO combinations posed challenges, often resulting in false positives where the model predicted success but clinical trials did not. For instance, pembrolizumab (anti-PD1) and vibostolimab (anti-TIGIT), with or without docetaxel, were predicted to be effective in metastatic NSCLC. The model rationale suggested that dual PD-1 and TIGIT blockade would reactivate T cells, while docetaxel would create a more immunogenic environment. However, in the phase 2 KeyVibe-002 trial, this combination failed to improve progression-free survival [20]. To reduce such mispredictions, incorporating more examples of failed trials is essential.

## 4. Discussion

Our approach significantly improves upon previous models not only by expanding the input training content on drug combinations, which, to date, have relied heavily on in vitro drug screens, but also by providing successful/failed studies in a clinical setting and an interpretable framework for both predictive power and biological insight. Unlike generic LLMs such as ChatGPT, which often generate inaccurate biological explanations, our model minimizes “hallucinations.” Direct comparisons to ChatGPT are challenging, as it lacks controls to prevent data leakage—where unseen drug combinations could still inform its responses. In contrast, our framework deliberately excluded test cases in the RAG to measure predictive accuracy accurately, a feature that general LLMs with full data access cannot replicate.

Recent LLM-based efforts like CancerGPT address cell-line synergy in few-shot settings; however, there remains no public, standardized benchmark for trial-level combination synergy against the framework that current study established.

Despite these promising results, limitations include lack of diversity in model training, such as that of drug modalities (e.g., CAR-T, cancer vaccines), ADC payloads (e.g., topoisomerase I inhibitor, microtubule inhibitor), and dosing considerations, and biases from published successful trials. We also acknowledge that potential factors, e.g., co-mutations, tumor microenvironment status, and specific prior therapy, can affect cancer progression and subsequent treatment outcomes, but in the current study these variables are treated as exploratory effect modifiers and not considered in the modeling unless supported by late-phase evidence in the clinical context. This choice limits generalizability outside the defined subcohorts but reduces the risk of over-interpreting early or hypothesis-level signals. Expanding these factors may improve accuracy.

In addition, our current validation cohort is modest (n = 58), which constrains precision of subgroup estimates and warrants continued accrual of endpoints. The current framework is intended to augment, not replace, clinical judgment. Ideally, deployment of this AI model requires a structured expert-review loop consisting of multidisciplinary adjudication of predictions with traceable evidence to further validate and refine outputs. Moreover, if deployed in practice, we will need to implement post-deployment monitoring and periodic recalibration updates aligned to indication/subcohort-specific outcomes.

In our current model, the weakest performance occurred for IO-IO pairs, exemplified by the framework’s false positive prediction for pembrolizumab + vibostolimab. Despite encouraging early signals and mechanistic plausibility, multiple late-phase readouts failed or were halted, including the program-wide discontinuation of vibostolimab combinations. The main reason for the false positive prediction is the data sparsity for IO-IO phase III trials and other factors that our framework did not capture, such as clinical biomarkers, antibody engineering variables, etc. In future work, to mitigate this, we will need to (1) recalibrate the prediction using negative-control phase III outcomes within IO-IO classes; (2) enrich features for IO biology and antibody properties; (3) down-weight relevant preclinical as well as early clinical evidence within the KG and RAG; (4) route IO-IO predictions through our expert-review loop if deployed in practice.

Despite these considerations, such predictive frameworks can ultimately accelerate the discovery of synergistic therapies, thereby reducing costly experiments, and improve overall patient outcomes in oncology.

## 5. Conclusions

This study presents a novel framework integrating LLMs with biological knowledge graphs to predict efficacy when combining 2–3 oncology drugs across a range of indications. By linking generative AI with explainability tools, we address two critical challenges in AI-driven drug discovery: predictive accuracy and mechanistic transparency. Such predictive models can accelerate the discovery of synergistic therapies, thereby reducing costly experiments, and potentially improve overall patient outcomes.

## Data Availability

The datasets used and analyzed during the current study are available from the corresponding author upon reasonable request.

## Supplementary Materials

The following supporting information can be downloaded at https://www.mdpi.com/article/10.3390/curroncol32100548/s1. 1. Figure S1: Distribution of drug modality combinations (left, N = 728) and total drug studies (right, N = 1631) used in the model. 2. Additional examples of synergy prediction using the model.

## Abbreviations

The following abbreviations are used in this manuscript:

LLM	Large Language Model
RAG	Retrieval-augmented generation
IO	Immuno-oncology
ADC	Antibody–drug conjugate
